# Regulation of Organic Hydroperoxide Stress Response by Two OhrR Homologs in *Pseudomonas aeruginosa*

**DOI:** 10.1371/journal.pone.0161982

**Published:** 2016-08-25

**Authors:** Sopapan Atichartpongkul, Paiboon Vattanaviboon, Ratiphorn Wisitkamol, Juthamas Jaroensuk, Skorn Mongkolsuk, Mayuree Fuangthong

**Affiliations:** 1 Laboratory of Biotechnology, Chulabhorn Research Institute, Lak Si, Bangkok, Thailand; 2 Applied Biological Sciences Program, Chulabhorn Graduate Institute, Lak Si, Bangkok, Thailand; 3 Center of Excellence on Environmental Health and Toxicology (EHT), Bangkok, Thailand; 4 Department of Biotechnology, Faculty of Science, Mahidol University, Bangkok, Thailand; 5 Center of Excellence on Emerging Bacterial Diseases, Faculty of Science, Mahidol University, Bangkok, Thailand; East Carolina University Brody School of Medicine, UNITED STATES

## Abstract

*Pseudomonas aeruginosa ohrR* and *ospR* are gene homologs encoding oxidant sensing transcription regulators. OspR is known to regulate *gpx*, encoding a glutathione peroxidase, while OhrR regulates the expression of *ohr* that encodes an organic peroxide specific peroxiredoxin. Here, we show that *ospR* mediated *gpx* expression, like *ohrR* and *ohr*, specifically responds to organic hydroperoxides as compared to hydrogen peroxide and superoxide anion. Furthermore, the regulation of these two systems is interconnected. OspR is able to functionally complement an *ohrR* mutant, i.e. it regulates *ohr* in an oxidant dependent manner. In an *ohrR* mutant, in which *ohr* is derepressed, the induction of *gpx* expression by organic hydroperoxide is reduced. Likewise, in an *ospR* mutant, where *gpx* expression is constitutively high, oxidant dependent induction of *ohr* expression is reduced. Moreover, *in vitro* binding assays show that OspR binds the *ohr* promoter, while OhrR binds the *gpx* promoter, albeit with lower affinity. The binding of OhrR to the *gpx* promoter may not be physiologically relevant; however, OspR is shown to mediate oxidant-inducible expression at both promoters. Interestingly, the mechanism of OspR-mediated, oxidant-dependent induction at the two promoters appears to be distinct. OspR required two conserved cysteines (C24 and C134) for oxidant-dependent induction of the *gpx* promoter, while only C24 is essential at the *ohr* promoter. Overall, this study illustrates possible connection between two regulatory switches in response to oxidative stress.

## Introduction

OhrR is an organic peroxide-sensing repressor that belongs to the MarR superfamily of transcription factors [1–3]. *Pseudomonas aeruginosa* OhrR has been shown to regulate *ohr*, which encodes a peroxidase that uses thiol and/or lipoic acid as cofactor [1, 4–7]. Deletion of *P*. *aeruginosa ohr* renders cells hypersensitive to organic peroxides [5]. The reduced form of OhrR binds to the *ohr* or *ohrR* promoters and represses their expression [1], but its oxidized form fails to bind the promoters [1]. The *P*. *aeruginosa* genome also contains an *ohrR* homolog, *ospR* [8]. The OhrR and OspR proteins share 46.5% identity. OspR has been shown to regulate the *gpx-ospR* operon [8]. Similar to an *ohr* deletion, mutation of *gpx* leads to a peroxide-sensitive phenotype [8]. OspR might also regulate other genes involve in antibiotic resistance and pigment synthesis [8].

Both OhrR and OspR belong to the two-cysteine subfamily of OhrR regulators. In *Xanthomonas campestris*, the ability of the OhrR repressor to sense organic hydroperoxide involves a key cysteine residue located at the N-terminus of helix α1 that acts as the sensor for organic hydroperoxides. Upon exposure of OhrR to organic hydroperoxides, the sensing cysteine residue is oxidized to a sulfenic acid intermediate that undergoes the rapid formation of a disulfide bond with the conserved cysteine residue located on the C-terminus [9]. Structural analysis shows that the oxidized form of OhrR undergoes a major conformational change, including the rotation of a winged helix that results in DNA disassociation [10]. This leads to the loss of the ability of the repressor to bind the promoter DNA and presumably allows RNA polymerase to bind the promoter and initiate the transcription of the detoxification enzyme gene [11–13]. Similarly, it has been shown that *P*. *aeruginosa* OspR also senses oxidant using a conserved cysteine at the N-terminus through sulfenic acid formation and intermolecular disulfide bond formation with another conserved cysteine at the C-terminus [8].

*P*. *aeruginosa* is a major cause of bacterial infections in patients with immuno-compromised conditions, cystic fibrosis and burns. As a defense mechanism against microorganisms, host cells produce reactive oxygen species, including superoxide anions and hydrogen peroxide (H_2_O_2_). Bacterial pathogens possess many antioxidant enzymes and regulatory systems, including OspR and OhrR, to protect themselves against these toxic reactive oxygen species. These regulatory systems probably have overlapping roles. Here, we described the connection between the OhrR and OspR systems in *P*. *aeruginosa* PAO1. The differential oxidant-sensing mechanism of OspR was also investigated.

## Materials and Methods

### Real-time polymerase chain reaction (qRT-PCR)

Total RNA was extracted from exponential phase cells (optical density at 600 nm [OD_600_] of ~0.4) cultured in Luria-Bertani broth using the modified hot acid phenol method [14]. The relative expression of specific transcripts was measured by qRT-PCR. All cDNA were prepared for qRT-PCR as previously described [1]. Briefly, contaminating DNA in the total RNA samples was hydrolyzed by the addition of DNaseI (Thermo Scientific) according to the manufacturer's instructions. The reverse transcription reaction was performed using a RevertAid^™^ M-MuLV Reverse Transcriptase kit (Thermo Scientific) according to the manufacturer's instructions using random hexa-primers. The real-time PCR reaction was performed using KAPA^®^ SYBR FAST (KAPA Biosystems) and StepOnePlus^™^ Real-Time PCR system (ABI, USA). The gene-specific primers were BT1554 (5′-GCACTCCGCGCGAACTGG-3′) and BT1555 (5′-CGGCAGGTTGATGTGCAG-3′) for *ohr*, BT204 (5′-TGCGGCTTACCCCGCAGTA-3′) and BT205 (5′-ACTTGGTGAAGTTCCACTT-3′) for *gpx*, and BT195 (5′-TGCTCTGGGAGTGGCATGC-3′) and BT196 (5′-ATCTCGTTGAGGTCGAAA-3′) for *gpxR*. The results were normalized to expression of 16s rRNA gene (BT2781 (5′-GCCCGCACAAGCGGTGGA -3′) and BT2782 (5′-ACGTCATCCCCACCTTCCT-3). All qRT-PCR runs included no template control reactions and DNA template control reactions. Specificity was verified by melting curve analysis and agarose gel electrophoresis (data not shown). The qRT-PCR reactions were performed in triplicate for each sample. At least 3 biological replicates were performed in all experiments. Ct value from each reaction was obtained. Ct value is the cycle number intersecting with the threshold line. The difference in Ct values of each gene (ΔCt) between the 16s rRNA gene and target gene was expressed as ΔCt = Ct_target_-Ct_16s rRNA_. The difference in ΔCt values (ΔΔCt) between treated sample and control sample was calculated. The point estimation of the expression ratio was calculated using 2^-ΔΔCt^.

### Determination of oxidant resistance levels

The exponential-phase cells were treated with oxidant at the indicated concentrations for 10 min. Washed cells were 10-fold serially diluted, and 10 μl of each dilution was spotted onto an LB agar plate. The plates were incubated at 37°C overnight before observation. At least 3 biological replicates were performed.

### Construction of the *ospR* mutant strain

The unmarked deletion of *ospR* in PAO1 was constructed using a Cre-*lox* system [15]. A 1.6-kp fragment containing *ospR* was amplified with the primers BT189 (5’-CTCAACAAGGAAGACAAG-3’) and BT1446 (5’-GGACTGTCTGATGGGACTGC-3’) and was cloned into pKNOCK-tet at the *Sma*I sites, yielding pKNOCK*ospR*. An *Eco*RI and *Eco*ICRI fragment containing the Gm resistance cassette flanked with *lox* sites from pCM357 [15] was gap-filled using Klenow fragment polymerase. This fragment was cloned into pKNOCK*ospR* at blunt-end *Pvu*II-*Pvu*II sites, yielding pKNOCKΔ*ospR*::*Gm*. The *Pvu*II digestion deleted 327 bp of the coding region of *ospR*. The pKNOCKΔ*ospR*::*Gm* plasmid was transformed into PAO1. To select for double cross-over events, gentamicin-resistant and carbenicillin-sensitive colonies were chosen. The *ospR* mutant with the antibiotic cassette replacement was then transformed with the pCM157 [15] vector containing the Cre-encoding gene. Cre, a site-specific recombinase, recognizes *lox* sites, and recombination between these sites deletes the DNA between the two sites. Transformants with the gentamicin cassette deletion or a gentamicin-sensitive phenotype contained the unmarked deletion of *ospR*. pCM157 was removed by growing cells under a nonselective condition. The deletion of *ospR* was confirmed by PCR and Southern blot analysis.

### Complementation plasmids

A 517-bp DNA fragment containing full-length *ospR* and a ribosome binding site was generated by PCR using the primers BT191 (5'-GATCGAGGCGCTGCTTGA-3') and BT192 (5'-GACGACGGCTAGCCTCCTA-3'). This fragment was first cloned into a broad-host-range vector, pBBR1MCS-5 [16], at the *Sma*I site to yield the plasmid pospR. The direction of fusion was checked by PCR to ensure that the gene was fused to the *Escherichia coli lacUV5* promoter present in the pBBR1MCS-5 vector. The insertions were checked by sequencing. Then, pospR was introduced into PAO1 strains by electroporation and selected for gentamicin resistance colonies.

### Primer extension

The primer used to detect the start site of *gpx* mRNAs was BT214 (5′- gtcttctgttcgccctt -3′), which is complementary to nucleotide sequences between +40 to +56, relative to the start codon of Gpx. Primer extension was performed as described previously [1]. A sequence ladder was generated using *fmol* DNA cycle sequencing system (Promega). The primer for sequencing was the same as used in primer extension reaction, and the template was a DNA fragment containing the promoter region.

### Purification of OspR

Primers BT192 and BT430 (5′ GCTTCCATGGGCACCCGGGGA 3′, *Nco*I site underlined) were designed and used in a PCR reaction with pOspR, pOspR-C24S, or pOspR-C134S as template. The purified *ospR* coding region digested with *Nco*I was ligated into the pETBlue-2 vector (Novagen) digested with *Nco*I and *Hin*cII to form the recombinant plasmid pET-OspR. The *ospR* gene and the promoter region were sequenced to ensure accuracy. This vector places the *ospR* gene under the control of the T7*lac* promoter and allows for the expression of *ospR* only in an *Escherichia coli* strain containing T7 RNA polymerase (BL21(DE3)). The pETBlue-2 vector also contains the *E*. *coli lac* operator in the opposite direction, allowing for blue/white screening for cloning. BL21(DE3) cells containing pET-OspR were grown in 200 mL Luria-Bertani broth containing 100 μg/mL ampicillin at 37°C on a rotary shaker until the cell density reached an OD_600_ of approximately 0.6–0.8. Then, IPTG (0.2 mM) was added to the culture, and growth was continued for 2 h. Cells were harvested by centrifugation, washed, and resuspended in KEGD buffer (25 mM KPO_4_, 2 mM EDTA, 5% glycerol, and 1 mM DTT) at pH 7.0. Cells were the disrupted using a French press. The lysates were clarified by centrifugation and loaded onto a DEAE column that had been equilibrated with the same resuspension buffer. The flow-through fraction was dialyzed in the KEGD buffer, pH 8.0, and then loaded onto a heparin sepharose column pre-equilibrated with KEGD buffer, pH 8.0. Non-absorbed materials were removed by passage of resuspension buffer through the column. OspR was gradient-eluted with KEGD buffer, pH 8.0, containing 50 and 500 mM KPO_4_. OspR was eluted at the appropriated salt concentration. After dialysis in KEGD buffer, pH 8.0, protein fractions were pooled, concentrated, and stored in KEGD buffer containing 20% glycerol. The purity of OspR was estimated to be at approximately 90% on a Coomassie blue stained SDS-polyacrylamide gel.

### DNaseI footprinting assay

The *gpx* promoter fragment was single end-labeled [γ-^32^P]ATP using T4 polynucleotidyl kinase. The fragment was 228 bp long and was produced by PCR using ^32^P-labeled BT 214 and BT462 (5′ CACCGGGTTGGGATCCTG 3′) primers. The *ohr* and *ohrR* promoter fragments were prepared using ^32^P-labeled BT 484 and BT468 primers and ^32^P-labeled BT 467 and BT483 [1] primers, respectively. The DNA binding reaction and DNaseI footprinting assay were conducted as previously described [1]. A sequence ladder was generated using *fmol* DNA cycle sequencing system (Promega). The template for the sequencing ladder was the corresponding promoter fragment itself, and the primer was the same ^32^P-labeled oligo used in the footprinting experiment.

### Gel shift assay

Promoter fragments were prepared and the DNA binding reaction was conducted as described above. Samples were then analyzed on a 5% polyacrylamide gel in 1x Tris borate-EDTA buffer containing 2.5% glycerol. The gel was dried and analyzed by autoradiography. The specific binding of OpsR to the *gpx* promoter fragment was proven by the inability of salmon sperm DNA at high concentration to compete with the low amount of *gpx* promoter fragment (data not shown).

### Site-directed mutagenesis of C24 and C134 of OspR

Site-directed mutagenesis was achieved via the PCR mutagenesis method described previously [1]. Primers containing mutational sites at the C24 and C134 were designed. The sequences of the primers used were as follows: BT396, 5′-ACCAGCTGTCTTTCAAGCTGT-3′ and BT397, 5′-ACAGCTTGAAAGACAGCTGGT-3′ for mutation of cysteine 24 into serine, and BT398, 5′-GCTGATCTCCAGCACCGGTTT-3′ and BT399, 5′ AAACCGGTGCTGGAGATCAGC 3′ for mutation of cysteine 124 into serine. The changed bases are underlined, and each pair was complementary to each other. The plasmids were sequenced and designated pospR-C24S and pospR-C134S.

### Promoter activity analysis

The *gpx* promoter-*lacZ* fusion was cloned into a broad host range expression vector. The 583-bp *gpx* promoter fragment was amplified using primers BT214 (5’-GTCTTCTGTTCGCCCTT-3’) and BT215 (5’- ATCGCGAGGTAGAGCCG- 3’) and cloned into the pDrive cloning vector (Qiagen). The pDrive vector with the *gpx* promoter inserted in the direction opposite to the *lacZ* cassette was chosen. The *gpx* promoter was excised from pDrive with *Bam*HI and *Eco*ICRI and directionally cloned upstream of the *lacZ* cassette of plasmid pUC18Sfi*lacZ* [1] digested with *Sma*I and *Bam*HI. The *Hin*dIII fragment from pUC18Sfi *Pgpx-lacZ* containing *gpx-lacZ* fragment was then cloned into the broad host range expression vector p027Ery [1] to produce p027*Pgpx-lacZ*. The promoter fragment was verified by sequencing. The plasmid was then introduced into *P*. *aeruginosa* strains by electroporation.

### β-galactosidase assay

Logarithmic phase cells were induced with oxidants for 30 min. After sonication, cell lysates were then used in the β-galactosidase assay [12]. At least three independent experiments were performed.

### Statistical analysis

Statistical analysis was performed using paired t-test. A *P* value of less than 0.05 was considered statistically significant.

## Results

### Specific induction of *gpx* and *ospR* transcription by organic hydroperoxides

**Fig 1:**
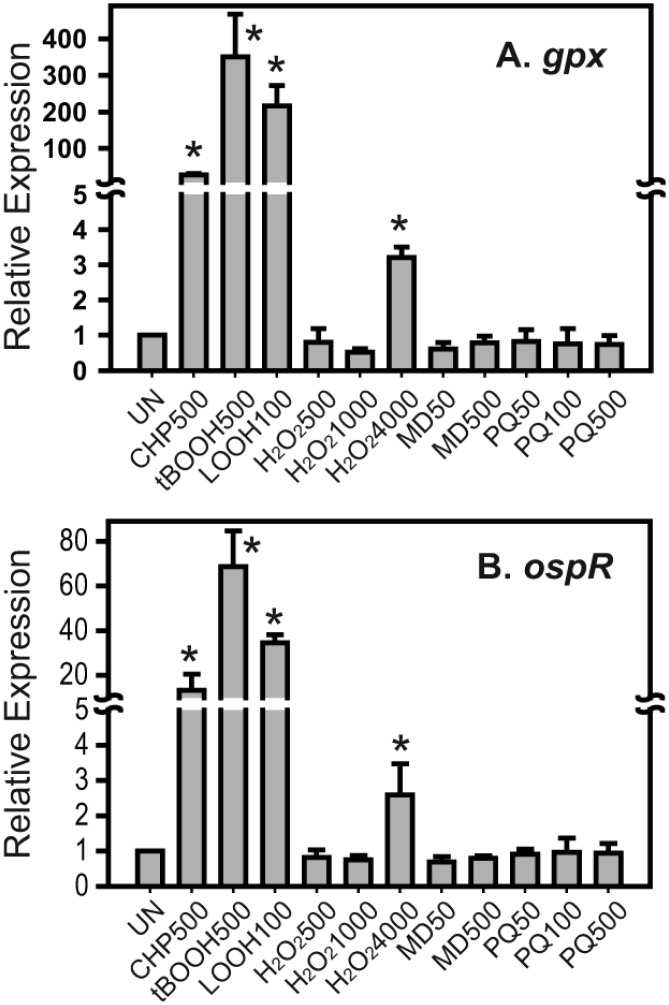
Expression analysis of *gpx* and *ospR* in the presence of oxidants. Quantitative real-time PCR was performed to measure relative expression levels (2^-ΔΔCt^) of *gpx* (A) and *ospR* (B) in wild-type *Pseudomonas aeruginosa* with various oxidant treatments for 15 min. The uninduced sample was set to 1. UN, uninduced; CHP500, 500 μM CHP; tBOOH500, 500 μM tBOOH; LOOH100, 100 μM linoleic hydroperoxide; H_2_O_2_500, 500 μM H_2_O_2_; H_2_O_2_1000, 1000 μM H_2_O_2_; H_2_O_2_4000, 4000 μM H_2_O_2_; MD50, 50 μM menadione; MD500, 500 μM menadione; PQ50, 50 μM paraquat; PQ100, 100 μM paraquat; PQ500, 500 μM paraquat. Significant differences (*P* < 0.05) between the treated samples and uninduced sample are denoted with asterisks.

The induction of the *gpx-ospR* (PA2826-PA2825) operon was monitored by quantitative real-time PCR analysis. Logarithmic cells of the wild-type *P*. *aeruginosa* strain were exposed to various oxidants for 15 min. Total RNA was extracted for real-time PCR analysis. Treatment of the wild-type strain with 500 μM cumene hydroperoxide (CHP), 500 μM *tert*-butyl hydroperoxide (tBOOH), and 100 μM linoleic hydroperoxide (LOOH) resulted in 27.6±4.1, 350.5±116.0, and 216.8±55.0-fold increases in the expression of *gpx* compared with the untreated sample, respectively (Fig 1A). Similarly, treatment of the wild-type strain with the same concentrations of CHP, tBOOH, and LOOH resulted in 13.3±7.1, 66.8±15.8, and 34.5±3.5-fold increases in the expression of *ospR* compared with the untreated sample, respectively (Fig 1B). These results indicate that both *gpx* and *ospR* show strong induction in the presence of organic hydroperoxides and lipid hydroperoxide (*P* < 0.05). In addition to lipid and organic hydroperoxides, we also treated *P*. *aeruginosa* with H_2_O_2_ (500 μM, 1000 μM and 4000 μM). The relative expression of *gpx* and *ospR* in cells treated with 4000 μM H_2_O_2_ was 3.2±0.3 and 2.6±0.9-fold higher than the control cells, respectively, while at 500 μM and 1000 μM H_2_O_2_, induction of *gpx* and *ospR* expression was not observed. Other reactive oxygen species were also tested, including the superoxide generators menadione (50 μM and 500 μM) and paraquat (50 μM, 100 μM and 500 μM) (Fig 1A and 1B). The results of these experiments showed that the relative expression levels of *gpx* and *ospR* in the presence of these superoxide generators in the treated samples were not significantly higher than the untreated samples (Fig 1A and 1B). The *katB* gene reported to be induced by oxidative stress including H_2_O_2_ and superoxide anion generator such as paraquat [17] was used to show that H_2_O_2_ and two superoxide anion generators i.e. paraquat and menadione used in this experiment were active. Relative expression of *katB* gene when induced with 500 μM H_2_O_2_, 1000 μM H_2_O_2_, 4000 μM H_2_O_2_, 500 μM menadione, and 500 μM paraquat was 40.5±4.9, 39.4±6.9, 37.8±16.3, 9.9±1.8, and 84.0±7.0-fold higher than the control cells, respectively, showing that reagents used were active (data not shown). This finding strongly suggests that *gpx* and *ospR* respond more specifically to organic/lipid hydroperoxides than H_2_O_2_ and superoxide anion.

### Role of *gpx* and *ospR* in peroxide resistance

**Fig 2:**
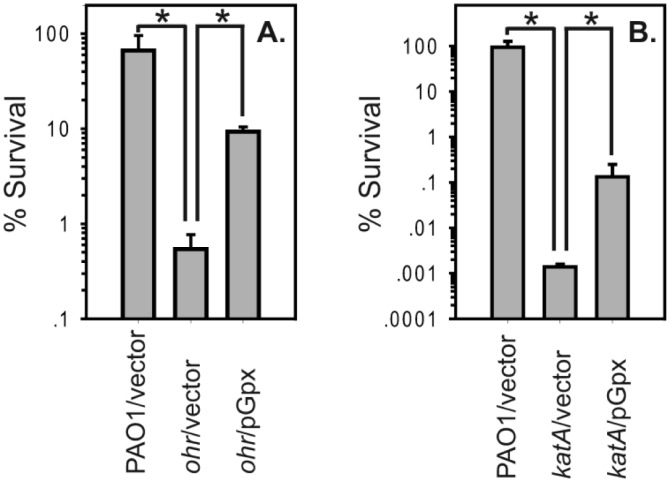
Gpx confers resistance to organic hydroperoxide and H_2_O_2_. (A) Percent survival of exponential phase *P*. *aeruginosa ohr* mutant containing pGpx treated with 1 mM tBOOH in comparison to the wild-type and the *ohr* mutant. (B) Percent survival of exponential phase *P*. *aeruginosa katA* mutant containing pGpx treated with 2.5 mM H_2_O_2_ in comparison to the wild-type and the *katA* mutant. Significant differences (*P* < 0.05) between samples are denoted with asterisks.

Next, we evaluated the function of *gpx* in *P*. *aeruginosa*. An unmarked deletion of *gpx* was constructed, and its hydroperoxides resistance was tested using cumene hydroperoxide, tert-butyl hydroperoxide, and hydrogen peroxide; however, no significant difference was observed when compared with the wild-type strain (data not shown). We hypothesized that this lack of peroxide sensitivity phenotype might be due to redundant peroxide detoxifications systems in *P*. *aeruginosa*. We then further investigated its possible function in *P*. *aeruginosa* strains with decreased ability to survive against organic hydroperoxides (*ohr* mutant) or H_2_O_2_ *(katA* mutant) [1, 5, 18, 19]. The *ohr* mutant containing the plasmid overexpressing the *gpx* gene showed increased resistance to *tert*-butyl hyderoperoxide (tBOOH), an organic hydroperoxide (Fig 2A). The organic hydroperoxide resistance level of the *ohr* mutant was two orders of magnitude less than that of the wild-type stain when cells were treated with 1 mM tBOOH. The overexpression of *gpx* partially restored the organic hydroperoxide-sensitive phenotype of the *ohr* mutant. Likewise, we found that the overexpression of Gpx partially restored the H_2_O_2_-sensitive phenotype of the *katA* mutant (Fig 2B).

### Expression of *ohrR* in the presence and absence of OspR

**Fig 3:**
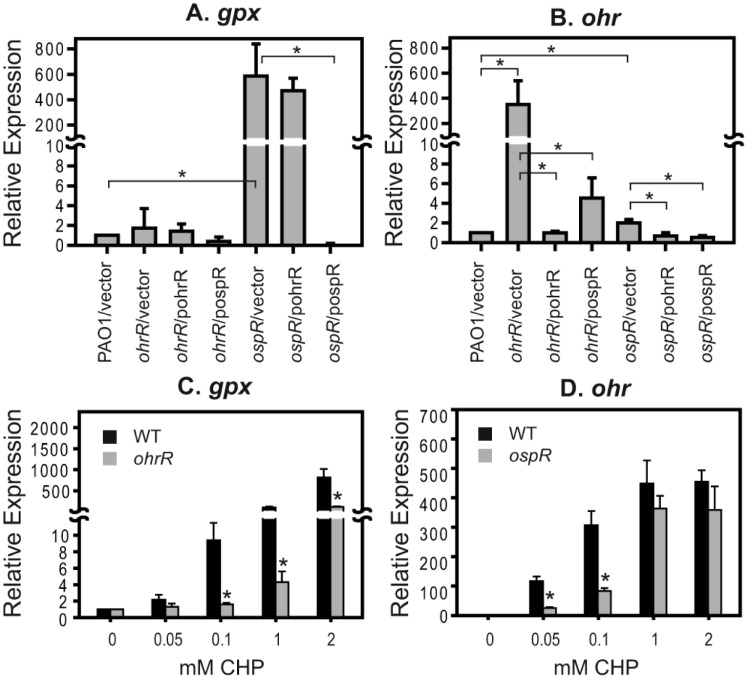
Regulation of *ohr* by OspR, the alteration of *ohr* expression in the *ospR* mutant and the alteration of *ospR* expression in the *ohrR* mutant. Quantitative real-time PCR was performed to measure relative expression levels (2^-ΔΔCt^) of *gpx* (A) and *ohr* (B) in various strains. (C) Expression of *gpx* in wild-type and *ohrR* mutant cultures treated with various concentrations of CHP (mM) for 15 min. (D) Expression of *ohr* in wild-type and *ospR* mutant cultures treated with various concentrations of CHP (mM) for 15 min. The expression in the wild-type strain was set to 1. Significant differences (*P* < 0.05) between samples are denoted with asterisks. For (C) and (D), means were compared between wild-type strain and mutant strain at the same condition.

Both OspR and OhrR belong to the MarR family of transcriptional regulators and have been shown to respond to oxidative stress, especially that caused by organic hydroperoxides [5]. We postulated that the function of OspR and OhrR might be connected. To test this hypothesis, a genetic approach was used. Substitution of OspR for OhrR and OhrR for *ospR* was performed in an *ohrR* mutant and an *ospR* mutant. A plasmid containing OhrR, termed pohrR, was introduced into the *ohrR* mutant [1] and the *ospR* mutant. Similarly, a plasmid containing OspR, termed pospR, was introduced into both the *ospR* mutant and the *ohrR* mutant. The expression of *ohr* and *gpx* was measured by quantitative real-time PCR to assess the function of OhrR and OspR. The level of *gpx* was predictably high in the *ospR* mutant (Fig 3A). When pospR was introduced into the *ospR* mutant, the expression level of *gpx* was approximately the same as that of wild-type cells. However, the level of *gpx* was high in the *ospR* mutant containing pohrR; this was similar to the pattern observed in an *ospR* mutant, indicating that OhrR could not function as a transcriptional repressor of the *gpx* gene (Fig 3A). As expected, *ohr* was up regulated in the *ohrR* mutant and was down regulated in the presence of a plasmid-borne functional copy of *ohrR* (Fig 3B) [1]. Surprisingly, complementation of the *ohrR* mutant with a functional copy of *ospR* led to the repression of *ohr* expression (Fig 3B). The expression of *ohr* in the *ospR* mutant was slightly up regulated (2.0±0.4 fold), and introduction of either *ohrR* or *ospR* into the *ospR* mutant suppressed the *ohr* expression (Fig 3B). These results lead us to speculate that OspR might be able to control the expression of *ohr* by binding to the *ohr* promoter.

### Alteration of *gpx* expression in the *ohrR* mutant and *ohr* expression in the *ospR* mutant during oxidative stress

In *P*. *aeruginosa* and other bacteria, *ohr* has been shown to play a crucial role in protecting cells from organic hydroperoxides [1, 5, 13, 20–22]. We have previously shown that the *ohrR* mutant had an increased level of organic hydroperoxide resistance in *P*. *aeruginosa*, and *ohr* expression was highly expressed in both reduced and oxidized conditions in the *ohrR* mutant [1]. We hypothesized that an *ohrR* mutant with an increased capacity to detoxify organic hydroperoxides might alter the expression of *gpx* in response to organic hydroperoxides. Expression analysis showed that when the *ohrR* mutant was exposed to various concentrations of CHP, the relative expression of *gpx* was statistically lower than that of the wild-type strain at 0.1 mM, 1 mM, and 2 mM CHP (Fig 3C). These results imply that CHP are readily detoxified by Ohr, and *gpx* is inducible by CHP at a concentration that will probably exceed the detoxification capacity of Ohr in the *ohrR* mutant. This is supported by the fact that the *ohrR* mutant expresses more *ohr* and is more resistant to organic hydroperoxide than the wild-type [1]. Furthermore, we observed a faster reduction of CHP in the *ohrR* mutant than the wild-type (supplement data). We also observed a change in the peroxide induction pattern of *ohr* in the *ospR* mutant. At 0.05 mM and 0.1 mM CHP, relative expression of *ohr* in the *ospR* mutant, where in the expression of *gpx* is high, was lower than that of the wild-type. At 1 mM and 2 mM CHP, a slight reduction in *ohr* relative expression was also observed in the *ospR* mutant, albeit not statistically significant (Fig 3D).

### Identification of OspR binding site at *gpx* promoter region

**Fig 4:**
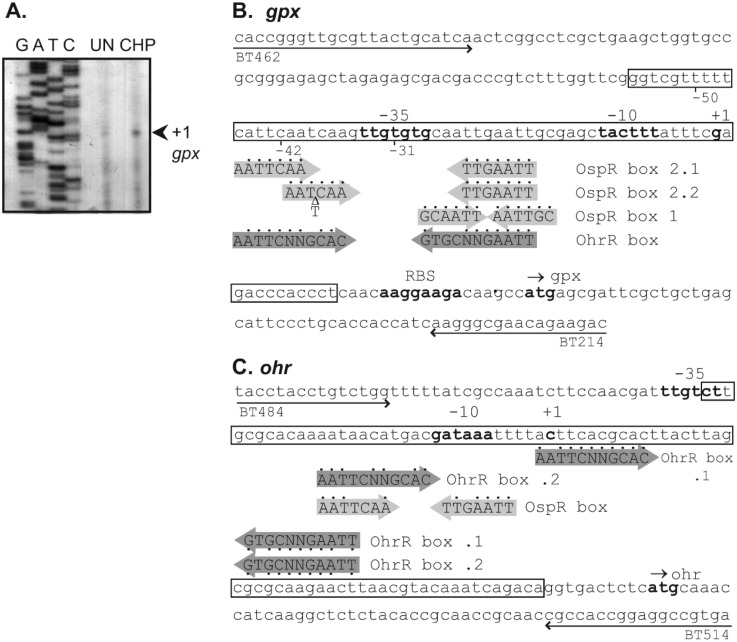
*ospR* and *ohr* promoter analysis. (A) Primer extension analyses of *gpx* using total RNA extracted from *Pseudomonas aeruginosa* without and with 200 μM cumene hydroperoxide induction. The arrow indicates the transcription start site. The Sanger sequencing ladder to the left of the primer extension lanes was generated using the same primer used in the primer extension. The nucleotide sequences of the *gpx* (B) and *ohr* (C) promoter regions are shown. The putative −10 and −35 promoter regions, the transcription start site (+1), and the putative translation start codon are shown in bold. Locations, directions and names of oligonucleotides used in this study, i.e. BT462, BT214, BT484, and BT514, are indicated. The DNaseI-protected regions for OspR binding are boxed. Putative OspR binding sites and OhrR binding site are aligned with the predicted conserved sequences in shaded arrows. Dots indicate matched nucleotides.

**Fig 5:**
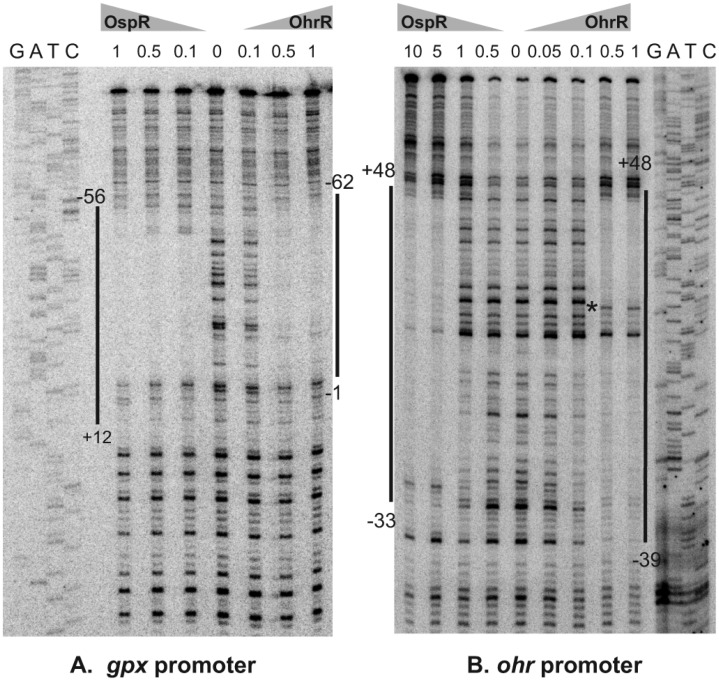
Mapping of the OspR binding sites on the *P*. *aeruginosa gpx* (A) and *ohr* (B) promoter fragments by DNase I footprinting. PCR-generated probe fragments were labeled on one strand by end-labeling one of the primers with ^32^P prior to amplification. The sequencing ladder (G, A, T, C) used to localize the binding sites on the promoters were generated using the promoter fragment itself as a template and the same labeled oligonucleotide as was used to generate the probe as a primer. Numbers above each lane indicate amounts of the OpsR and OhrR protein (μM) used in each reaction. The regions protected by OspR or OhrR are indicated by vertical lines. Hypersensitive site is indicated by asterisk.

Although putative OspR binding sites and promoter sequences were previously reported [8], these have not yet been experimentally verified. We first performed a primer extension assay to determine the transcription start site of *gpx*. RNA samples prepared from uninduced and 200 μM CHP-induced wild-type cultures were used. An 86-bp primer extension product was detected in the CHP-induced RNA sample. This placed the *gpx* transcription start site 30 bp upstream from the *gpx* translation initiation codon (Fig 4A). This information allowed us to predict the putative -10 (TACTTT) and -35 (TTGTGTG) boxes of the *gpx* promoter (Fig 4B), which concur with previously predicted -10 and -35 sequences [8]. To determine the DNA sequence bound by OspR at the *gpx* promoter region, a DNaseI footprinting experiment was performed using the purified OspR protein. As seen in Figs 4B and 5A, the region of the *gpx* operator protected by OspR extended from nucleotides +12 to -56, with respect to the *gpx* transcription start site.

**Fig 6:**
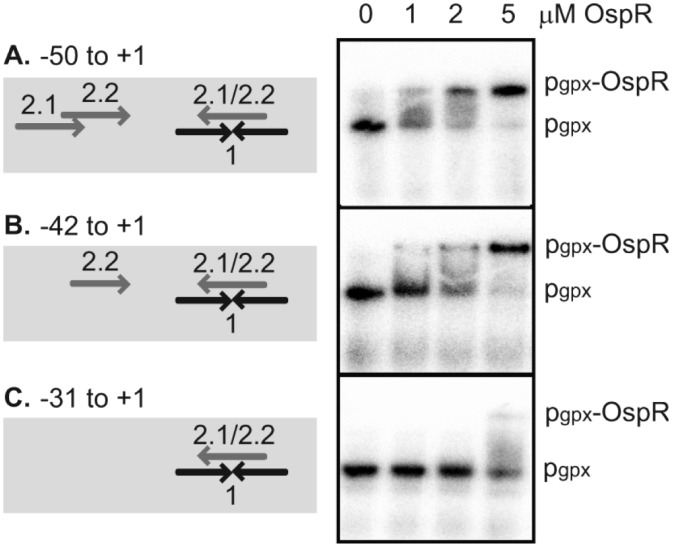
Mapping of the OspR binding sites on the *Pseudomonas aeruginosa gpx* promoter region. Gel shift assay of purified OspR protein with various *gpx* promoter fragments. The concentrations of OspR protein added in the binding reactions are indicated above each lane. The unbound promoter fragment was designated p_gpx_, and the protein-DNA complex was designated p_gpx_-OspR.

The DNA binding site of the OhrR protein family is proposed to involve an inverted repeat with a core sequence of AATT-N-AATT (N is any nucleotide, and the number of nucleotides is variable) [11, 13, 20, 22, 23]. Inspection of the *gpx* operator region revealed more than one possible OspR binding site within the DNaseI-protected region of the *gpx* operator. A previously predicted site, GCAATT-N-AATTGC (OpsR box 1), is a perfect inverted repeat [8] and is identical to the *Streptomyces coelicolor* OhrR binding site (Fig 4B) [22]. We proposed an additional conserved binding sequence, AATTCAA-N-TTGAATT (OspR box 2.1 and 2.2) that also contains the core sequence of AATT-N-AATT (Fig 4B). To gain insight into which inverted repeat is the OspR binding site, we carried out gel shift assay using three different *gpx* operator fragments. The first fragment (nucleotides -50 to +1) contains all proposed putative OspR binding sites (Figs 4B and 6A). The second fragment (nucleotides -42 to +1) contains OspR boxes 1 and 2.2. The third fragment (nucleotides -31 to +1) contains the entire OspR binding box 1 but only half of box 2 (Fig 4B). The binding affinity of OspR to fragment 1 appeared to be slightly higher than its affinity to fragment 2 (Fig 6A and 6B). While up to 5 μM, OspR only slightly shifted the *gpx* operator fragment that contains a deletion of one half of box 2 but retained a complete box 1 (Fig 6C). These results showed that box 1 alone was not sufficient for OspR binding, suggesting that box 2 is more likely the OspR binding site at the *gpx* operator.

### Analysis of the OspR operator sequences in the *ohr* promoter region

Next, we identified the binding site of OspR on the *ohr* promoter region. A DNaseI footprinting reaction containing OhrR and the *ohr* promoter fragment was performed as a control reaction. In agreement with previous reports, OhrR binding protected the *ohr* promoter fragment at nucleotides -39 to +48, with respect to the transcription start site of *ohr* (Fig 5B) [1]. Binding of OspR to the *ohr* promoter fragment created a DNaseI-protected region slightly shorter than the binding of OhrR. The protected region of OspR on the *ohr* operator region extended from nucleotide position -33 to +48 (Fig 5B). However, the binding patterns of the two proteins were significantly different. The OspR binding at the *ohr* promoter did not yield a hypersensitive site as was observed in the OhrR binding at the *ohr* promoter.

Surprisingly, OhrR also bound the *gpx* promoter at the same region that OspR bound (Fig 5A). The OhrR binding region on the *gpx* promoter covered nucleotides -1 to -62 compared with nucleotides +12 to -56 bound by OspR on the same *gpx* promoter fragment (Fig 5A). In addition, the footprint at *gpx* promoter was observed at a higher concentration of OhrR than OspR (500 nM vs 100 nM, respectively, in this experiment).

### Differential requirement of cysteines for oxidant sensing of OspR at the *gpx* and *ohr* promoters

**Fig 7:**
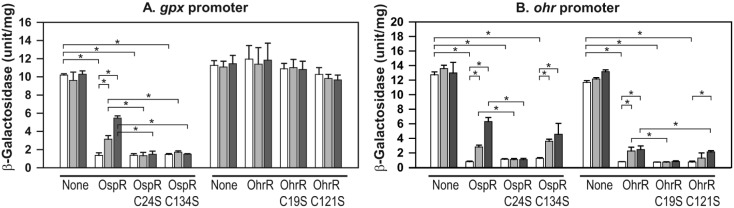
Effects of cysteine residues of OspR on the *gpx* promoter activity. Overnight cultures of *ospRohrR* double mutant and complementation strains carrying the *gpx-lacZ* reporter fusion (A) or the *ohr-lacZ* reporter fusion (B) were grown to mid-log phase and collected for *β*-galactosidase assays. Experiments were performed three times; error bars represent the standard error of the mean. White, light gray and gray bars represent control culture with no induction, sample with 500 μM cumene hydroperoxide induction and sample with 100 μM linoleic hydroperoxide induction, respectively. Significant differences (*P* < 0.05) between samples are denoted with asterisks.

The deduced amino acid sequence of OspR exhibited two conserved cysteine residues at position 24 and 134. It has been shown *in vitro* that C24 is important for oxidative sensing through sulfenic acid formation and then intermolecular disulfide bond formation with C134 [8]. In this study, we aimed to confirm the role of these two cysteines in the peroxide-sensing mechanism *in vivo*. We constructed serine substitutions of the OspR cysteines (OspRC24S and OspRC134S). These two *ospR* mutants and the wild-type *ospR* gene were cloned into a broad-host-range expression vector, pBBR1MCS-5, and transformed into a *P*. *aeruginosa ospRohrR* double*-*mutant strain that contains a *gpx-lacZ* reporter fusion. As expected, the *ospRohrR* double*-*mutant displayed constitutively high expression of β-galactosidase activity, and the plasmid-borne copy of wild-type OspR successfully complemented this phenotype (Fig 7A). When pospRC24S was introduced into the *ospRohrR* mutant, β-galactosidase activity was observed at a low level, indicating that OspRC24S repressed the expression of *gpx*. Thus, OspRC24S maintained DNA binding activity (Fig 7A). However, induction by either CHP or LOOH was not apparent (Fig 7A). Like all other members of OhrR, this result strongly suggests that C24 plays a role in the oxidant sensing of *P*. *aeruginosa* OspR [1, 12, 20]. Surprisingly, OspRC134S complementation showed a CHP induction pattern similar to that observed with the OspRC24S complementation (Fig 7A). The OspRC134S mutant protein retained DNA binding activity but did not seem to be able to sense the presence of CHP or LOOH. This finding indicates that both C24 and C134 are important for the oxidant sensing ability of OspR at the *gpx* promoter.

We further investigated the oxidant sensing mechanism of OspR at the *ohr* promoter. First, when pohrR, pohrRC19S, and pohrRC121S were introduced into the *ospRohrR* double-mutant containing an *ohr-lacZ* reporter fusion, the same pattern of β-galactosidase activity was observed as previously reported using RT-PCR (Fig 7B) [1]. The double mutant containing pohrR could lower the β-galactosidase activity and retained the ability to be induced by organic hydroperoxide. The introduction of pohrRC19S into the double mutant resulted in a constitutive low level of β-galactosidase activity (Fig 7B). This is expected because C19 is likely to play a role in the oxidant sensing ability of *P*. *aeruginosa* OhrR, while the introduction of pohrRC121S in the double mutant showed similar β-galactosidase activity as the double mutant strain containing pohrR (Fig 7B). The pospR, pospRC24S or pospRC134S plasmids were then introduced into a *P*. *aeruginosa ospRohrR* mutant strain that contains an *ohr-lacZ* reporter fusion. In the presence of pospR, β-galactosidase activity produced by *ohr-lacZ* reporter fusion was repressed, and CHP and LBOOH treatments resulted in the production of elevated levels of β-galactosidase activity (Fig 7B). When pospRC24S was introduced into the double mutant, we observed a low level of β-galactosidase activity produced by *ohr-lacZ* in both untreated and organic hydroperoxide-treated conditions, emphasizing the role of C24 in redox sensing (Fig 7B). We found that the introduction of pospRC134S into the *ospRohrR* mutant resulted in the repression of *ohr-lacZ* expression. Unlike OspRC24S, OspRC134S retained the peroxide-inducible pattern at the *ohr* promoter (Fig 7B).

## Discussion

*Pseudomonas aeruginosa* possesses two organic hydroperoxide resistance regulators, OhrR and OspR, that belong to the OhrR super family of transcriptional regulators. In this study, we investigated the possible overlapping roles of these two oxidation-sensing transcriptional regulators. *P*. *aeruginosa* OhrR regulates the *ohr* gene that encodes the peroxiredoxin or thiol-dependent peroxidase that catalyzes the reduction of hydroperoxides, especially organic hydroperoxides [1, 7]. *P*. *aeruginosa* OspR regulates many genes, including *gpx* (glutathione peroxidase gene), which is located upstream of *ospR* [8]. Glutathione peroxidase reduces hydroperoxides using electrons from glutathione or thioredoxin. Both H_2_O_2_ and organic hydroperoxides are generally known to be substrates of glutathione peroxidase [24, 25]. Our data suggest that overexpression of *P*. *aeruginosa* Gpx confers both H_2_O_2_ and organic hydroperoxide resistance in a *P*. *aeruginosa katA* and *ohr* mutant, respectively (Fig 3). The H_2_O_2_ detoxification ability of *P*. *aeruginosa* Gpx concurs with a previous report when Gpx was overexpressed in the wild type strain [8]. Therefore, it is conceivable that both Ohr and Gpx protect *P*. *aeruginosa* from oxidative stress.

Similar to the *ohrR/ohr* genes from other bacteria [13, 22, 23, 26], *P*. *aeruginosa ohrR/ohr* transcripts are highly inducible by organic hydroperoxides [1]. It has been shown that high concentrations of H_2_O_2_ upregulate the transcription of *ospR* [8]. Here, we demonstrated that exposing *P*. *aeruginosa* to organic hydroperoxides, including 500 μM cumene hydroperoxide, 500 μM tert-butyl hydroperoxide, and 100 μM linoleic hydroperoxide, led to the induction of *gpx* and *ospR* transcripts (Fig 1). Concentrations of linoleic hydroperoxide as low as 10 μM induced the expression of *gpx* (data not shown), and exposure of *P*. *aeruginosa* to 4 mM H_2_O_2_ slightly elevated the expression of *gpx* and *ospR* (Fig 1). In addition, the overexpression of *gpx* complemented the organic hydroperoxide-sensitive phenotype of the *P*. *aeruginosa ohr* mutant (Fig 2). These results strongly suggest that both OhrR and OspR play a role in protecting *P*. *aeruginosa* from organic hydroperoxides.

This hypothesis is further supported by the observation that high expression of OspR in the *ohrR* mutant or the *ohrRospR* mutant almost completely suppressed *ohr* expression (Figs 3 and 7). The binding of OspR to the *ohr* promoter was shown by DNaseI footprinting analysis (Fig 5), suggesting that, in addition to OhrR, OspR could be a putative transcriptional regulator of *ohr*. The expression of *ohr* in the *ospR* mutant was slightly higher than that of the wild-type strain; a 2-fold induction was observed (Fig 3B). We reasoned that the overexpression of Ohr protein in the *ohrRospR* double mutant or the *ohrR* mutant masked the effect of OspR on *ohr* expression because high Ohr could rapidly detoxify organic hydroperoxides [13, 23]. This phenomenon is also shown in Fig 3. The induction level of *gpx* in the *ohrR* mutant where *ohr* expression is very high was significantly lower than in the wild-type strain where *ohr* expression is much lower [1]. These data were confirmed by promoter-fusion assays (data not shown). Furthermore, the affinity of OspR to the *ohr* promoter appeared to be lower than the affinity of OhrR to the same *ohr* promoter fragment (Fig 5B). On the other hand, the overexpression of OhrR in the *ospR* mutant or the *ohrRospR* mutant did not suppress the expression of *gpx* (Figs 3 and 7). Surprisingly, we observed the binding of OhrR at the *gpx* promoter, albeit at a lower affinity (Fig 5A).

The DNA binding sequence of OhrR homologs has been shown to be an AT-rich motif [13, 20, 22, 23]. The *P*. *aeruginosa* OhrR box, AATTCNNGCAC-N-GTGCNNGAATT, also contains AATT-N-AATT [1]. Both OhrR and OspR bind the *ohr* and *gpx* promoters *in vitro* (Fig 5). Previously, it has been proposed that the GCAATT-N-AATTGC sequence at the *gpx* promoter is the OspR-binding motif, and it has been shown that AATT-N-AATT sequence of this motif is essential for OspR binding (Fig 4B) [8]. However, in this study, we showed that this GCAATT-N-AATTGC is not sufficient for OspR binding on the *gpx* promoter (Fig 6). Therefore, we proposed a new OspR binding site, AATTCAA-N-TTGAATT (Fig 4). Half of this new OspR binding site overlaps with the previously proposed binding site [8]. This might explain why the deletion of AATT-N-AATT at the *gpx* promoter abolished OspR binding [8]. At the *gpx* promoter, the OspR binding sites were mapped to 5'-cATTCAA-N_15_-TTGAATT-3' (box 2.1) and 5'-AATCAA-N_11_-TTGAATT-3' (box 2.2) (Fig 4B). Capital letters indicate the nucleotides that match the conserved binding site. There is only one mismatch for OspR box 2.1 at the first nucleotide of the conserved motif and one deletion for OspR box 2.2. At the *ohr* promoter, there are 5 mismatches, 5'-AATaacA-N_5_-aTaAATT-3' (Fig 4C). It has been shown experimentally that OspR also binds the *hmgA* and *PA1897* promoters [8], and putative OspR boxes can be identified on these promoters. The OspR box at the *hmgA* promoter contains 3 mismatches, 5′-AATTCAA-N_10_-cgtAATT-3′. The OspR box at the *PA1897* promoter contains 3 mismatches (5′-AATTtAA-N_12_-TTtcATT-3′). The sequences protected from DNaseI by OhrR or OspR are very similar at both the *gpx* and *ohr* promoters. However, the affinity appears to be different. For the *gpx* promoter, the binding affinity of OspR is higher than OhrR. For the *ohr* promoter, the binding affinity of OhrR is higher than OspR. The difference in the binding affinity might be biologically relevant and may dictate which protein is the major regulator of each gene.

There are two major subfamilies of OhrR based upon the number of key cysteine residue at the reactive site: one-cys OhrR and two-cys OhrR [2, 3]. The one-cys OhrR prototype is *Bacillus subtilis* OhrR [11, 27]. Other examples of the one-cys OhrR are *Streptomyces coelicolor* OhrR [22], *Staphylococcus aureus* MgrA [28], and *S*. *aureus* SarZ [29]. This one-cys OhrR subfamily uses its conserved N-terminal cysteine to sense peroxide. The oxidized cysteine becomes sulfenic acid as intermediate and then reacts with small molecules thiols such as bacillithiol in *B*. *subtilis* to form a mixed-disulfide bond [11, 27]. The two-cys OhrR was first identified in *Xanthomonas campestris* [9]. Similar to the one-cys OhrR, the conserved N-terminal cysteine of the two-cys OhrR acts as oxidant sensor. The conserved N-terminal cysteine is oxidized to sulfenic acid and then forms an intramolecular disulfide bond with the less conserved C-terminal cysteine. Both *P*. *aeruginosa* OhrR and OspR belong to the two-cys-type OhrR subfamily. Their N-terminal cysteines are essential for oxidant sensing (Fig 7) [1, 8]. For *X*. *campestris* OhrR, the C-terminal cysteine is essential for the conformational change of OhrR that leads to the inactivation of its DNA binding activity [9, 10]. On the other hand, *P*. *aeruginosa* OhrR does not require C121 for its peroxide sensing mechanism [1], suggesting that C121 plays a role in preventing the overoxidation of the redox-sensing cysteine [30]. In *Chromobacterium violaceum*, the C126 mutation of OhrR partly affects its oxidative sensing mechanism [26]. Interestingly, we reported here that the essentiality of the C-terminal cysteine for the oxidant-sensing ability of OspR depends on the promoter it reacts with. OspR requires C134 for full oxidant sensing ability at the *gpx* promoter (Fig 7). In contrast, the requirement of this C-terminal cysteine for the oxidant sensing at the *ohr* promoter is much lower (Fig 7). This result provides insight into how a transcriptional regulator may differentially regulate genes according to differences in the target DNA. It has been demonstrated that as little as a single base pair difference in the binding site of the glucocorticoid receptor could lead to differences in protein conformation and its regulatory activity [31]. The importance of C-terminal cysteine of OhrR in the oxidative-sensing mechanism and DNA binding activity remains to be elucidated.

The biological reason that *P*. *aeruginosa* maintains two OhrR homologs is not well understood. Gene duplication is quite common in bacteria and other organisms [32, 33]. It is believed that there are many benefits to gene duplication and divergence, including facilitation of adaptation and evolution [33, 34]. It has been demonstrated that transcription factors could have the same, overlapping or different group of target genes [34]. According to our knowledge, OspR directly regulates additional genes apart from its own regulon, for example, the *hmgA* genes involved in amino acid metabolism [8]. It is possible that the gene duplication of OhrR homologs leads to the functional diversification of these transcription factors to fine-tune the regulatory network or to benefit cells in response to different environmental insults. Therefore, one hypothesis is that OspR and OhrR work together to control gene expression in a hierarchical manner. OspR might be a generalized oxidative stress regulator that represses both *ohr* and *ohrR* expression. Once exposed to oxidative stress, OspR loses DNA binding ability leading to the expression of *ohrR*. However, *ohr* is still repressed at this point. In the event that organic peroxide accumulates, OhrR becomes inactive and the more specialized Ohr can detoxify organic peroxides. This hypothesis is partially supported by the fact that OspR also binds the *ohrR* promoter albeit at low affinity (unpublished data).

## Supporting Information

S1 Fig The ability of the *ohrR* mutant and the wild-type *Pseudomonas aeruginosa* to reduce cumene hydroperoxide. The FOX Assay was modified and used to quantify the ability of *Pseudomonas* strains to diminish organic hydroperoxide (Chuchue T. et al. 2006. *ohrR-ohr* are the primary sensor/regulator and protective genes against organic hydroperoxide stress in *Agrobacterium tumefaciens*. J.Bacteriol. 188:842–51). The overnight cultures were subcultured to OD_600_ = 0.05 in 60 mL LB medium. Cells were grown to mid-log phase (OD_600_ = 0.4). Cell pellet was resuspended in 400 μL Phosphate Buffer Saline (PBS) on ice and sonicated. After centrifugation to remove cell debris, supernatant was kept on ice. 600 μg total protein of each cell lysate was incubated with 100 μM CHP in 500 μL reaction volume, at 37°C with shaking. PBS without cell lysate and containing 100% CHP was used as control. The aliquots of 100 μL were taken out at different time points (2, 5, 10 and 15 min) to react with 900 μL FOX reagent (25 mM H_2_SO_4_, 100 μM Fe(NH_4_)_2_ (SO_4_)_2_, 125 μM Xylenol-Oragnge) for 10 min. The remaining amount of peroxide was measured at 540 nM. Error bars represent standard deviation of three biological replicates.
(PDF)
