# Supplementary figures and images for: Regulation of Organic Hydroperoxide Stress Response by Two OhrR Homologs in Pseudomonas aeruginosa
Source: PLoS One. 2016 Aug 25;11(8):e0161982. doi: 10.1371/journal.pone.0161982 (PMC4999210; doi:10.1371/journal.pone.0161982)

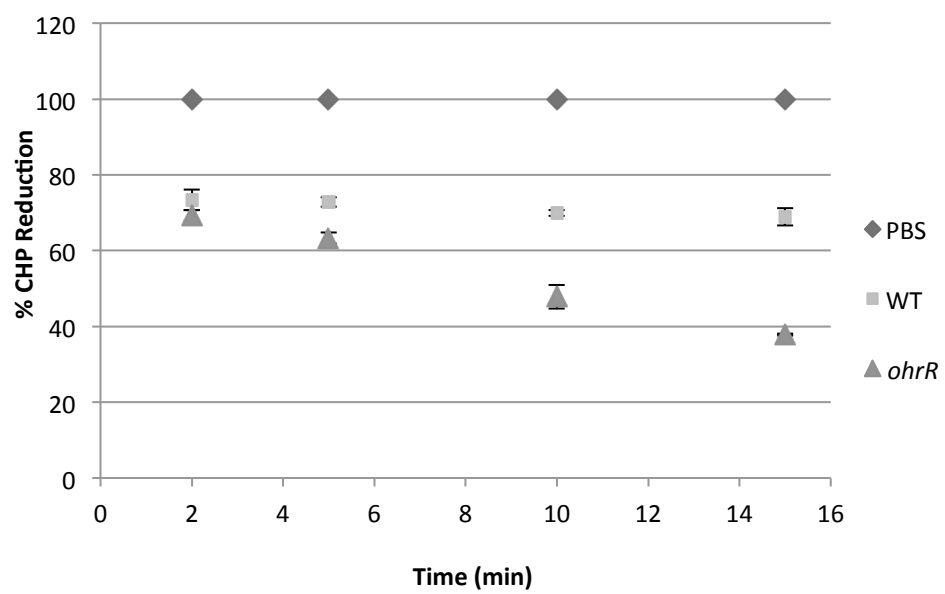

**S1 Fig**

Supplement: S1 Fig — The FOX Assay was modified and used to quantify the ability of Pseudomonas strains to diminish organic hydroperoxide (Chuchue T. et al. 2006. ohrR-ohr are the primary sensor/regulator and protective genes against organic hydroperoxide stress in Agrobacterium tumefaciens. J.Bacteriol. 188:842–51). The overnight cultures were subcultured to OD600 = 0.05 in 60 mL LB medium. Cells were grown to mid-log phase (OD600 = 0.4). Cell pellet was resuspended in 400 μL Phosphate Buffer Saline (PBS) on ice and sonicated. After centrifugation to remove cell debris, supernatant was kept on ice. 600 μg total protein of each cell lysate was incubated with 100 μM CHP in 500 μL reaction volume, at 37°C with shaking. PBS without cell lysate and containing 100% CHP was used as control. The aliquots of 100 μL were taken out at different time points (2, 5, 10 and 15 min) to react with 900 μL FOX reagent (25 mM H2SO4, 100 μM Fe(NH4)2 (SO4)2, 125 μM Xylenol-Oragnge) for 10 min. The remaining amount of peroxide was measured at 540 nM. Error bars represent standard deviation of three biological replicates. (PDF) [file pone.0161982.s001.pdf]
